# Starch-Based Polymer Materials as Advanced Adsorbents for Sustainable Water Treatment: Current Status, Challenges, and Future Perspectives

**DOI:** 10.3390/polym15143114

**Published:** 2023-07-21

**Authors:** Pui San Khoo, R. A. Ilyas, M. N. A. Uda, Shukur Abu Hassan, A. H. Nordin, A. S. Norfarhana, N. H. Ab Hamid, M. S. A. Rani, Hairul Abral, M. N. F. Norrrahim, V. F. Knight, Chuan Li Lee, S. Ayu Rafiqah

**Affiliations:** 1 Centre for Advanced Composite Materials, Universiti Teknologi Malaysia, Skudai 81310, Johor, Malaysia; puisankhoo@utm.my (P.S.K.); shukur@utm.my (S.A.H.); 2 Faculty of Chemical and Energy Engineering, Universiti Teknologi Malaysia, Skudai 81310, Johor, Malaysia; abuhassannordin@gmail.com (A.H.N.); farahfarhana.as@gmail.com (A.S.N.); 3 Institute of Tropical Forest and Forest Products (INTROP), Universiti Putra Malaysia, Serdang 43400 UPM, Selangor, Malaysia; iker.asmal55@gmail.com (M.S.A.R.); chuanli@upm.edu.my (C.L.L.); ayurafiqah@upm.edu.my (S.A.R.); 4 Centre of Excellence for Biomass Utilization, Universiti Malaysia Perlis, Arau 02600, Perlis, Malaysia; nuraiman@unimap.edu.my; 5 Faculty of Mechanical Engineering and Technology, Universiti Malaysia Perlis, Arau 02600, Perlis, Malaysia; 6 Faculty of Mechanical Engineering, Universiti Teknologi Malaysia, Skudai 81310, Johor, Malaysia; 7 Department of Physics, Faculty of Science, Universiti Putra Malaysia, Serdang 43400 UPM, Selangor, Malaysia; 8 Laboratory of Nanoscience and Technology, Department of Mechanical Engineering, Andalas University, Padang 25163, Indonesia; abral@ft.unand.ac.id; 9 Research Collaboration Center for Nanocellulose, BRIN-Andalas University, Padang 25163, Indonesia; 10 Research Centre for Chemical Defence, Universiti Pertahanan Nasional Malaysia, Kem Perdana Sungai Besi, Kuala Lumpur 57000, Malaysia; victor.feizal@upnm.edu.my

**Keywords:** starch, adsorbent, wastewater treatment, heavy metals, dye, oil, organic solvents, pesticides, pharmaceutical pollutants, micropollutants

## Abstract

Over the past three decades, chemical and biological water contamination has become a major concern, particularly in the industrialized world. Heavy metals, aromatic compounds, and dyes are among the harmful substances that contribute to water pollution, which jeopardies the human health. For this reason, it is of the utmost importance to locate methods for the cleanup of wastewater that are not genuinely effective. Owing to its non-toxicity, biodegradability, and biocompatibility, starch is a naturally occurring polysaccharide that scientists are looking into as a possible environmentally friendly material for sustainable water remediation. Starch could exhibit significant adsorption capabilities towards pollutants with the substitution of amide, amino, carboxyl, and other functional groups for hydroxyl groups. Starch derivatives may effectively remove contaminants such as oil, organic solvents, pesticides, heavy metals, dyes, and pharmaceutical pollutants by employing adsorption techniques at a rate greater than 90%. The maximal adsorption capacities of starch-based adsorbents for oil and organic solvents, pesticides, heavy metal ions, dyes, and pharmaceuticals are 13,000, 66, 2000, 25,000, and 782 mg/g, respectively. Although starch-based adsorbents have demonstrated a promising future for environmental wastewater treatment, additional research is required to optimize the technique before the starch-based adsorbent can be used in large-scale in situ wastewater treatment.

## 1. Introduction

The Sustainable Development Goals (SDGs) [1] are a set of 17 global goals that aim to promote sustainable development in economic, social, and environmental ways. Among the listed SDG, SDG 6 is to ensure everyone has access to and can manage water and sanitation in a sustainable way. This makes water quality issues a priority at the international level. However, water pollution is still a major challenge to achieving SDG 6 and other goals for sustainable development. According to the World Health Organization (WHO) [2], at least 2 billion people around the world consume contaminated water. This can cause health problems like diarrhea, cholera, and typhoid fever. Additionally, water pollution has big effects on ecosystems and biodiversity, leading to reduced aquatic life, the loss of wetland areas, and damage to coastal zones. Water pollution causes economic and social problems, such as making it harder for people and communities to obtain clean water. It is clear that fixing water pollution is a key part of ensuring everyone has a good future [3,4,5,6].

Studies show that more than a billion people around the world do not have access to clean water, and it is thought that water pollution is the cause of about 2.2 million deaths in developing countries [7,8]. These significant numbers show how important it is to take action to solve the global water crisis and provide clean water for human beings [9]. Water that has already been used in some capacity is considered wastewater, such as water from houses or effluent from factories [10]. Human activities such as mining and agriculture have caused water pollution, but rapid urbanization has made the situation worse, as enormous amounts of wastewater have been discharged into the environment without treatment [11,12,13]. Water resources in these circumstances must be constantly protected because misuse could be harmful to humans. Chemical, biological, or physical contaminants may be present in wastewater. It might be dangerous for human use as a result. If untreated wastewater enters the public water system, it may result in serious disease [14,15]. There are several pollutants such as heavy metal ions and pharmaceutical pollutants which cause serious water pollution all around the world [13]. It is therefore important to create a variety of effective technologies for the removal of contaminants from wastewater due to stringent legislation on the release of these harmful products.

In the past, certain industry factories did not prioritize wastewater treatment due to the high cost of setting up and operating treatment plants, as well as the lack of strict legislative enforcement regarding effluent discharge. However, there has been a shift in some countries towards a more serious approach, with stricter legislation governing industrial effluent. For instance, China implemented the Water Pollution Control Plan in 2015, which requires businesses and industries to establish wastewater treatment facilities for pollutant removal and water reuse [16,17]. Similarly, the European Nation (EN) introduced the EU Water Frame Directive in 2000, following a similar approach [18,19]. These measures aim to address the environmental impact of industrial wastewater by mandating the implementation of wastewater treatment facilities to remove pollutants and promote water reuse.

During the past three decades, several physicochemical, biological, and advanced technologies have been used for wastewater treatment such as flotation, precipitation, oxidation, biodegradation, advanced oxidation process, and others [20,21]. Researchers are now investigating new wastewater pollution removal technologies and materials relevant to sustainability. Considering the pressure on climate change and the needs in the clean water, sustainable wastewater treatment is necessary to meet our needs [22]. In contrast to conventional methods, adsorption technologies are crucial for the elimination of hazardous pollutants because of their great efficiency, extensibility, and sensitivity to harmful compounds [23]. In general, adsorption is a technique for treating wastewater that can be used to remove several chemicals from industrial effluent. Adsorption takes place when liquid molecules adhere to the surface of a solid substance. As a matter of fact, natural biopolymer-based adsorbents that are less expensive have generated a lot of academic interest [24,25].

Starch is a carbohydrate and a natural component of most plants, is commercially derived from grains and serves as an important raw material in various industries such as medicine, food, chemicals, etc. Starch has been extensively studied for its potential in wastewater treatment [26,27], it is a relatively good option for wastewater treatment due to its chemical structure, biocompatibility, and biodegradability which can enhance its utilization as green adsorbent. Cassava starch, rice starch, corn starch, and potato starch are documented botanical sources of starch [28]. Starch molecules exist in two structural forms: amylose and amylopectin. Amylopectin contains a higher glucose content compared to amylose [29]. In its natural state, amylose accounts for about 20–30% of starch and amylopectin accounts for 70–80%. Starch is primarily synthesized in the chloroplast of plant leaves or the amyloplast of plant storage organs and contains lipids as well as phosphate groups [30]. However, native starch has considerable limitations when used in wastewater treatment. These limitations include low surface area, limited thermal stability, low water solubility, low molecular weight, quick degradability in water, and a lack of reactive functional groups [31,32]. To overcome these limitations and enhance its adsorption capabilities for wastewater treatment, researchers have explored various modifications of starch. Researchers discovered that incorporating a chemical functional group into the starch backbone improves the adsorption efficacy of modified starch to a variety of pollutants [31,32,33]. Several starch modifications, including starch-based grafts, polymer nanocomposites, nanofibers, nanoparticles, activated carbon, biochar, hydrogels, aerogels, and beads, have been developed to overcome these limitations [31]. Through modification and functionalization approaches, ongoing research aims to improve the adsorption capacity and selectivity of starch-based adsorbents.

The utilization of starch-based materials for environmentally friendly water treatment is an increasingly explored area of research. In this comprehensive review, we aim to provide a detailed overview of the current advancements in starch-based materials, including their extraction from biomass, their suitability as adsorbents, and their potential applications in wastewater treatment. The review will delve into the properties of these materials and emphasize their distinctive advantages in the realm of wastewater treatment. The central focus of this review is on sustainable water treatment, shedding light on how starch-based materials can be utilized after modification to address the most pressing environmental challenges, and how their implementation in wastewater treatment can contribute to the attainment of the Sustainable Development Goals. Moreover, this review will provide valuable insights into the adsorption capabilities of starch-based materials and their most promising uses in removing various pollutants such as oil, organic solvents, pesticides, heavy metals, dyes, pharmaceutical pollutants, and more. By exploring the potential of starch-based materials in treating various types of pollutants, we aim to provide a comprehensive understanding of their efficacy and potential impact in wastewater treatment. Overall, this review aims to serve as a valuable resource for researchers and professionals in the field of water treatment, providing a comprehensive analysis of the current state of starch-based materials and their potential in sustainable wastewater treatment practices.

## 2. Adsorption

Adsorption is a robust process in which adsorbate (gases, liquids, or solutes) adhere to the surface of the adsorbent [34], reducing the effluent volume to a minimum [35]. Adsorption of adsorbate to adsorbent surfaces can happen both physically (physisorption) and chemically (chemisorption) [36]. The interaction between the adsorbate and adsorbent are weak chemical bonds and physical forces, such as functional group changes, hydrogen bonding, electron donor acceptor, and electrostatic interaction. In water and wastewater treatment industries, adsorption is chosen due to its cost-effectiveness and dependability in comparison to other processes. Adsorption is found to be the best strategy because it is efficient, moderately simple to operate, economical, and generates few by-products. It is also extremely industrially advantageous due to its superior regeneration capabilities [37]. In addition, its uses are vast and varied, ranging from the preparation of drinkable water to the removal of non-biodegradable organic substances originating from groundwater. With its numerous advantages, it is not surprising that adsorption is widely used in the water and wastewater treatment industries. With its ability to selectively remove certain contaminants from water and its comparatively low cost, adsorption offers a possible solution to the pressing problems of water pollution and sustainability. As researchers continue to investigate new and innovative applications of adsorption in water treatment, it is anticipated that this process will play an ever-increasing role in protecting the health of our communities and the world [38,39].

### 2.1. Adsorbents

Adsorbents are the materials that can perform adsorption itself. These materials come in the form of porous solids with a large surface area [40]. Adsorbent selection is critical to the success of any adsorption-based water treatment process. The criteria that need to be considered to choose a material for an adsorbent is from its cost-effectiveness, availability, sustainability, suitable mechanical properties, does not disintegrate in solution, longevity, regeneration, etc. As to its performance, an adsorbent’s performance is dependent on the physical structure, activation conditions, influence of process variables, solution conditions, and the chemistry of its pollutants [41].

**Figure 1:**
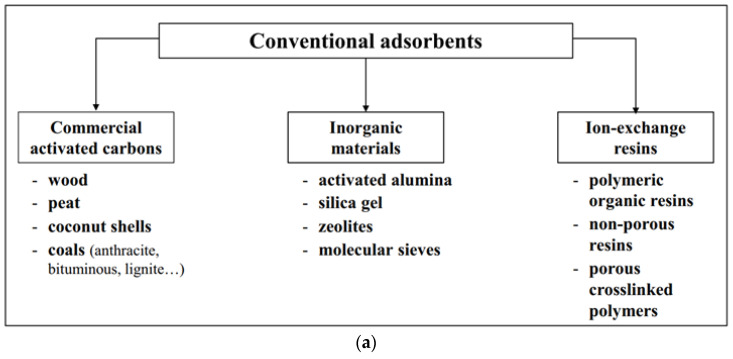

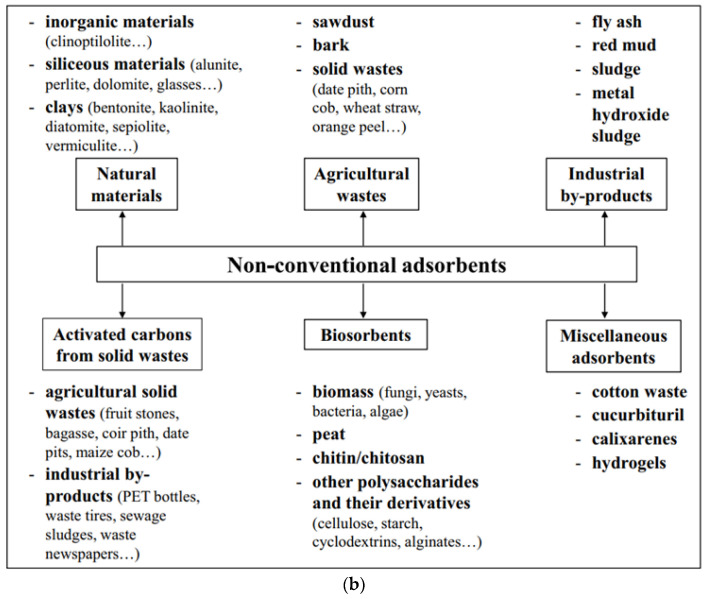
(**a**) Conventional adsorbents and (**b**) non-conventional adsorbents for removal of pollutants from wastewaters [44].

There are five different categories that can distinguish one adsorbent from another. These categories are natural materials, manufactured materials, modified natural materials, agricultural solid wastes and industrial by-products, and bio-sorbents. These then can be divided again into two different, much more simplified groups. Conventional adsorbents and non-conventional adsorbents. Conventional adsorbents include activated carbons, such as wood, peat, coals, coconut shells; inorganic materials, such as silica gel, natural zeolites, activated alumina, and molecular sieves, which are synthetic zeolites; and ion-exchange resins, such as polymeric organic resin, non-porous resins, and porous cross-linked polymers. As for non-conventional adsorbents, these include activated carbons from solid wastes; bio-sorbents, such as chitosan, cellulose, starch, and biomass; industrial by-products; agricultural wastes; natural materials such as clays, siliceous materials, and inorganic materials; and some miscellaneous adsorbents such as cotton waste, hydrogels, etc. [42,43]. Figure 1 presents the examples of conventional adsorbents and non-conventional adsorbents for removal of pollutants from wastewaters.

Several ways of utilizing non-conventional adsorbents have been investigated in order to develop greener, cheaper, and highly effective adsorbents to adsorb pollutants [44]. There has been a surge in the development of non-conventional adsorbents, especially those derived from biopolymers such as cellulose [45,46], chitosan [47,48], starch, and their derivatives, because they are abundant and readily available, non-toxic, low-cost, biodegradable, and renewable [49,50]. These biopolymers are intriguing owing to their physicochemical properties, high reactivity, chemical structure stability, and selectivity towards functional groups [51,52].

### 2.2. Starch-Based Adsorbents

Starch, the most abundant available biopolymer in the biosphere, is a homopolysaccharide with linear and branched units derived from a variety of sources, including tuber waste [53,54,55,56]. It is extensively utilized in the food industry as a source of energy in human diets, and it has discovered usages as a raw material in non-food industries for instance water treatment [57]. Native starch, on the other hand, has significant disadvantages such as low thermal stability, low surface area, slight water solubility, low molecular weight, rapid degradability in water, and a lack of reaction functional groups, which inhibit it from being utilized as a wastewater treatment adsorbent. As a result, starch must be modified to improve its adsorption efficiency towards various pollutants by changing its chemical surface structure via incorporating functional groups into the starch backbone [31,32].

Starch is composed of numerous hydroxyl groups that are easily modified by the incorporation of functional groups, such as primary amine, carboxylic or sulfonic acid to produce starch-based grafts, hydrogels and beads, polymer nanocomposites, aerogels, nanofibers, and so on, to improve its adsorption capacity for wastewater treatment [31,58]. Owing to the presence of a carboxymethyl functional group, starch derivatives, particularly carboxymethyl starch (CMS), have received a great deal of attention in both research and industry [57]. Starch that has been functionalized, grafted or crosslinked with amine [59,60], carboxylic [61], and carboxymethyl [62] groups along its chains, giving it a high affinity for heavy metal ion removal.

Conventional adsorbents, such as activated carbon, are considered good adsorbents due to their large surface area and excellent adsorption property [63], but starch-based adsorbents have several advantages in the aspects of adsorption capacity, desorption capacity, and lifetime. Starch-based adsorbents have been demonstrated to have a high adsorption capacity for both organic and inorganic pollutants from wastewater when compared to conventional adsorbents owing to their porosity and the presence of many functional groups. Adsorption capacity of starch-based adsorbents can be increased not only by modifying them with functional groups but also by combining them with other materials. For instance, Li et al. [53] developed hydrogel microspheres by crosslinking hydroxyethyl starch and carboxymethyl chitosan with epichlorohydrin for removal of heavy metal ions and dye. In another study, starch-functionalized magnetic Fe_2_O_3_ has been shown to adsorb up to 2000 mg/g of lead ions from polluted water at 150–450 mg/L lead ions concentration [64], whereas activated carbon functionalized magnetic iron oxide nanoparticles has a lower adsorption capacity of 61.82 mg/g at 10–100 mg/L lead ions concentration [65]. Table 1 compares the adsorption capacities of starch-based and conventional adsorbents.

Aside from adsorption capacity, desorption efficiency is critical for reusing utilized starch-based adsorbents. According to Gunawardene et al. [76], modified starch has a desorption efficiency of more than 97%. Starch-based adsorbents are renewable and can be regenerated by washing them with reagent. Reagents for desorption include sodium hydroxide (NaOH) [77], hydrochloride acid (HCl) [77], acetone [78], and more. On the other hand, conventional adsorbents, such as activated carbon, need to be replaced periodically due to their limited lifetime. The use of activated carbon is limited due to its regeneration issues, high cost and not environmentally friendly during its production [63].

In terms of durability and longevity, conventional adsorbents and properly maintained starch-based adsorbents can degrade/decompose in a few months to a few years. However, the degradation rate of starch-based materials is accelerated at high temperatures and high humidity levels, reducing their performance [79]. Extreme operating conditions, such as high humidity, harsh chemical environments, high temperatures, or pressure, will reduce the lifetime of starch-based materials even further [80,81]. In addition, starch-based materials are easily attacked by soil microorganisms and degrade rapidly [82]. Junlapong et al. [83] developed cassava starch hydrogels adsorbents that were 80% degraded after 30 days when buried in soil. The duration of biodegradation and decomposition of starch-based adsorbents is affected by several factors, including adsorbent composition, operating conditions, and the presence of microorganisms.

### 2.3. Starch-Based Adsorbents for In situ and Ex Situ Water Remediation

Starch-based adsorbents can be used in the adsorption stage of water treatment. Various adsorbents will be applied at this stage to remove pollutants from the wastewater via adsorption mechanism. Starch-based adsorbents derived from cornstarch [84,85,86,87], rice flour [88], cassava starch [83,89,90], graham flour [88], and potato starch [84,85,91,92] are safe compounds with the potential to be used for in situ water remediation. Starch-functionalized iron oxide nanocomposite, for example, has been utilized to adsorb heavy metal ions from wastewater produced from different industries [64]. The adsorption efficiency of Cd(II) from tap, marine, and industrial wastewater samples ranged from 87 to 93%, 84 to 91%, and 76 to 90%, respectively, whereas the adsorption efficiency of Hg(II) ranged from 69 to 93%, 40 to 89%, and 70 to 94%, respectively. Pb(II) had the maximum adsorption efficiency of 97 to 98%, 92 to 97%, and 93 to 98% for tap, marine, and industrial wastewater samples. Additionally, these starch-based nanocomposites have the potential to be a sustainable adsorbent for metal ions removal from industrial wastewater, marine water, and tap water with 76 to 93%, 70 to 94%, and 93 to 97% of percentage recovery, respectively [64]. Table 2 provides examples of starch-based adsorbents used for in situ water treatment. Comparing wheat starch with and without methanol pretreatment, the adsorption efficiency increased from 20.50% to 32.72% with methanol pretreatment. However, corn starch pretreated with methanol has a lower adsorption efficiency than corn starch without pretreatment [93]. In another investigation conducted by Kim et al. [94], a facultative psychrophilic denitrifier (strain 47) was immobilized on macro-porous cellulose carriers, and soluble starch was utilized as an electron donor to remove nitrate contaminants from groundwater resources. Groundwater temperature was found to be an important factor in affecting nitrate removal effectiveness in in situ water remediation. At a hydraulic retention time of one hour, nitrate removal efficiency of 99.5% can be attained. These findings emphasize the importance of considering factors such as pretreatment methods and environmental conditions when utilizing starch-based adsorbents for in situ water remediation.

The optimum conditions for ex situ water remediation using starch-based adsorbents will vary depending on the type of pollutant, structure (functional group, chain length, etc.) of the starch-based adsorbent, adsorbent dose, pollutant concentration, pH of the treating bath, treatment duration, and agitation speed [35]. Starch-based adsorbents have been found to be effective at removing pollutants at concentrations ranging from a few milligrams per liter to several hundred milligrams per liter [61], as reported in Table 1. The initial pollutant concentration is a driving force in overcoming the mass transfer resistance of pollutants between water and adsorbent [95]. Nevertheless, the effectiveness of starch-based adsorbents may decrease at higher pollutant concentrations due to adsorbent surface saturation, increasing the electrostatic repulsion force between the saturated adsorbent and adsorbate in the aqueous solution [96]. Other factors, such as reaction time, pH, temperature, and competing ions in the water, may also have an impact on the performance of starch-based adsorbents [95,97].

In this case, the adsorbent of interest is starch, one of the non-conventional “green” adsorbents that are highly effective at removing a wide variety of pollutants from wastewater. They have higher adsorption capacities and longer lifetimes than conventional adsorbents. Additionally, they are highly advantageous compared to the conventional adsorbents due to their physiochemical nature, abundance, and relatively inexpensive pricing, showing their effectiveness in pollutant adsorption in water treatment processes [98,99]. Furthermore, their renewability and biodegradability make them an appealing alternative to conventional adsorbents, promoting sustainable and eco-friendly industrial process solutions.

## 3. Chemical Structure and Properties of Starch

### Chemistry and Properties

**Figure 2:**
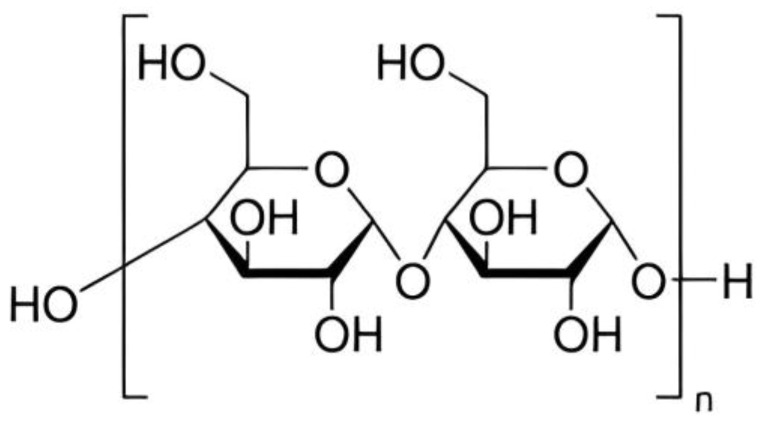
Chemical structure of starch composed of glucose molecules [108].

Starches with basic chemical formula, ${\left(C_{6}H_{10}O_{5}\right)}_{n}$ is a carbohydrate naturally found in many grains and vegetables, such as wheat, maize, and potatoes [100,101]. Starch is a part of polysaccharide which exists in two structural forms: amylose and amylopectin [102,103]. One of the most abundant polysaccharides in nature, starch provides us with a good supply of additional carbs [104]. The size of starch granules varies, ranging from the sub-micron elongated chloroplast granules to the comparatively enormous oval granules of potato and canna [105,106]. Biosynthesis of starch is a complex process [101,106]. It is also composed of glucose molecules [107] as shown in Figure 2.

**Figure 3:**
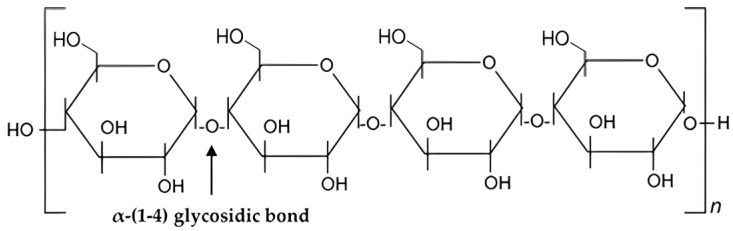
Chemical structure of amylose.

Amylopectin and amylose are parts of starch that are layered and packed in semi-crystalline and amorphous layers in concentric growth rings [109]. Amylose, which predominantly contributes to the amorphous phase, is a basically unbranched (0.1–0.5% branched) polymer made up of α-(1–4)-linked glucose units [110]. Since amylopectin has more α-(1–6) branch points, it has a more branched structure [110]. Amylose, one of the structural forms of starch, exists as a glucose bonded together in a linear chain or helical chain. Amylose also does not dissolve in water [107]. Figure 3 shows the chemical structural of amylose.

Amylose has an interesting solution property: it can form complexes with iodine and some organic reagents [111]. The amylose/cellulose nanofiber combination has numerous exceptional qualities, including strong heat resistance and storage stability [110]. Starch has a relative amount of amylose which varies according to its source. For example, corn starch has about 28 wt.% compared to cassava starch, with 17 wt.% [112].

**Figure 4:**
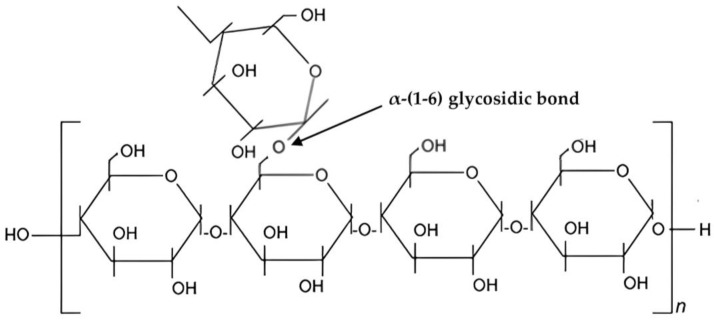
Chemical structure of amylopectin.

Amylopectin is the other structural form of starch. Depending on the botanical origin, the amylose percentage of starch granules varies, although conventional cereal starches typically include between 20 and 30 percent amylose [113]. The ability to control the functions of starch depended on the production of lengthy branch-chains of amylopectin [114]. The ability of long branch-chains of amylopectin to self-assemble structured structures and form complexes with lipids or sodium palmitate resulted in a considerable reduction in starch viscosity and slowing of starch digestion [114,115,116]. Amylopectin is a linear chain of glucose molecules, and it contains a much larger amount of glucose compared to amylose [107]. The structural form of amylopectin can be seen in Figure 4.

The weakly bonded linkages between bonding molecules make amylopectin a water-soluble component. Amylopectin is responsible for the crystalline properties of starch. Since amylose is a minor component of starch, amylopectin is the counterpart of amylose. Amylopectin is the major component of starch by weight and one of the largest molecules found in nature [117].

## 4. Applications for Water Treatment

Current environmental degradation on a global scale presents a serious problem for contemporary society. Industrialization, urbanization, agricultural methods, and human activities are all contributing to significant increases in pollution levels in the environment. These contaminants have the potential to have devastating effects on human health, animal health, and ecosystem health. Negative health effects may occur after these substances are ingested, absorbed, or inhaled [118,119]. Additionally, persistent organic contaminants in biota and fish, as well as the bioaccumulation of several heavy metals along the food chain, pose a major hazard to people and wildlife [19]. To detect, monitor, and remove dangerous pollutants, effective, affordable, and sustainable methods are required.

While starch can be used as one method of sustainable water treatment, native starch has low solubility in organic solvents and low adsorption ability [50,120]. The highly active hydroxyl groups on the backbone of starch can be modified by different functional groups (amino, carbonyl, carboxyl, ester, etc.) using various reaction routes like grafting, cross-linking, esterification, oxidation, and irradiation to increase its adsorption efficacy towards dyes [50,121], heavy metals [122], phenols [123], etc. Phosphorylation, followed by grafting of polymethacrylic acid onto native starch, produces phosphate and carboxylic functional groups on the starch backbone, leading to a higher tendency to make hydrogen bond with phenol [123]. In order to increase the solubility of corn and potato starches, they were modified via the acetylation method [84]. The acetylation of starch increases swelling power and solubility by weakening the bond strength between amylose and amylopectin molecules [124], conforming to the starch microparticle and favoring electrodynamic mechanisms for flocculation processes [84].

Various modified starches, like cationic starches, carboxymethyl starches, starch phosphate, starch xanthate, starch sulphate, starch carbamate, carboxyl methyl starch and so on, are cheap and have been used as efficient adsorbents for remediation over the past 10–15 years [50,122]. The modified starch-based adsorbents, such as starch-based composites and nanoparticles and starch-based hydrogels, are more useful due to their large surface area, available polar sites, and reproducibility in the degree of activation [50]. Type, botanical origin, branching/networking, chain length, granule size, degree of depolymerization, and applied modification (enzymatic, physical, chemical, etc.) all affect the desired properties of starch [125]. Starches also can be modified from activated carbon starch to starch nanocrystals and chitosan–starch nanocomposites. Other various of starch related can also be made for the adsorption process are HMS-SiO_2_@MSC, mesoporous activated carbon from starch (ACS), MNPs@Starch-g-poly(vinyl sulfate) nanocomposite, silica-sand/anionized-starch composite (CMS-SS), and Starch-Mg/Al layered double hydroxide (S-Mg/Al LDH).

Additionally, β-cyclodextrin (β-CD), a naturally occurring cyclic oligosaccharide consisting of seven glucose units, has emerged as a highly promising adsorbent for the removal of organic micropollutants from contaminated water [126]. β-CD is derived from starch through enzymatic conversion and has demonstrated remarkable efficacy in the removal of various organic micropollutants in recent years. Dyes [127], pesticides [127], metals [126], and pharmaceuticals such as ibuprofen [128,129], naproxen [130], imipramine, bisphenol-S, procaine, ciprofloxacin [126], acetaminophen [131], salbutamol, atenolol [132], estradiol (E2) [133], and 17β-estradiol [134] have all been successfully removed using β-CD-based adsorbent. β-CD has hydroxyl groups on the exterior of the torus, making it hydrophilic, while the interior is hydrophobic [135]. As β-CD has good solubility in water, it cannot be directly used in water treatment. Nevertheless, by cross-linking β-CD with a suitable cross-linking agent, a β-CD-based adsorbent can be developed [136,137,138]. In fact, there are few types of cyclodextrin (CD): alpha-cyclodextrin (α-CD), beta-cyclodextrin (β-CD), and gamma-cyclodextrin (γ-CD). The cavity volume of α-CD is approximately 17.4 nm^3^, the cavity volume of β-CD is approximately 26.2 nm^3^, and the cavity volume of γ-CD is approximately 25.6 nm^3^. These cavity volumes control the size and shape of the cavity within each cyclodextrin molecule, which influences their ability to encapsulate pollutants of varied sizes and shapes [139]. These findings contribute valuable insights into the design and manufacturing of effective adsorbents from starch for wastewater treatment. The utilization of β-CD as a potential adsorbent material opens new possibilities in the field of water treatment, particularly for the removal of organic micropollutants.

### 4.1. Removal of Oil and Organic Solvent

There is a significant amount of concern about different contaminants inside the contaminating water, including traditional pollutants like heavy metals and organics, as well as developing micropollutants, personal care products, and substances that interfere with hormones. For purging toxins from wastewater, several researchers are investigating more sustainable materials and methods. The industries that produce chemical extraction, petrochemistry, textiles, and food all produce large amounts of oily wastewater, and this wastewater’s excessive discharge has significantly endangered both human health and the environment. These problems can be successfully overcome with starch-based adsorbents for oil and organic solvent removal. Table 3 shows examples of starch-based adsorbents for oil and organic solvent removal.

**Figure 5:**
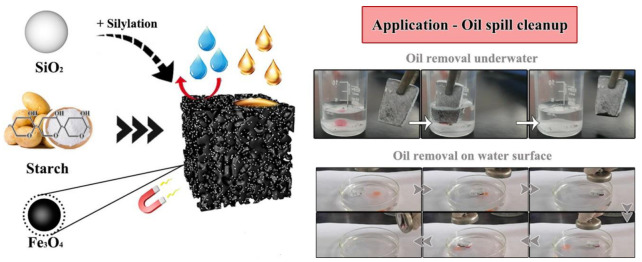
Application of superhydrophobic and magnetic starch-based adsorbent to remove oil [141].

By incorporating nanoparticles into starch cryogel, Wang et al. [141] created a brand new super-hydrophobic sorbent, also known as HMS-SiO_2_@MSC, that may be utilized to clean up oil spills (Figure 5). HMS-SiO_2_@MSC showed good viability in cleaning the oil slick magnetically and by removing oil underwater. The material’s extraordinary ability to absorb oil is in part due to the barrier of trapped air where the chemical inertness of covalent bonding (Si-O-C/Si), and the structural support provided by pore walls. Pore-rich starch or Fe_3_O_4_ is being mixed with micro-/nanoparticles (SPF@SC), developed in another study by Wang et al. [144], which was intended to remove occasional oil patches. To clean up occasional oil slicks, magnets remotely control SPF@SC.

**Figure 6:**
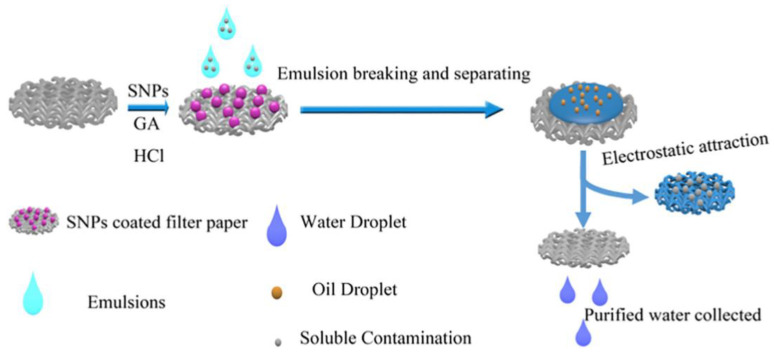
Application of SNPs for removing oil [147].

There are also variety of other methods to remove oil from the wastewater, for example, gravity sedimentation, and oil absorption by biomaterials. In contrast to emulsions stabilized by surfactants, immiscible oil or water mixtures are what these approaches are meant to separate. Membrane separation, which in this context is an advantage of the sieve effect, is widely recognized as being an effective method of handling emulsified oil or water mixtures. However, its extensive use is constrained by the unavoidable fouling caused by oil droplets or absorbed surfactants. Using pH-responsive SNPs as the coating material, starch, which is also referred to as a biological macromolecule, is employed as biodegradable super hydrophilicity and underwater superoleophobicity filter paper in a low-cost, easy, and eco-friendly manner. The SNPs were only coated and adhered to the filter paper’s surface. The improved filter paper has switchable super hydrophilicity and underwater superoleophobicity wetting behavior due to its hierarchical structure. The filter paper worked well as designed when used to filter out oil from water and oil in water emulsions at the same time. Additionally, the filter paper displayed a unique pH-sensitive quality as well as exceptional stability and recyclable qualities. Above all, the filter paper covered with starch-based nanospheres offers a creative way to separate challenging oil-in-water emulsions [147]. Figure 6 shows the application of SNPs for oil removal.

### 4.2. Removal of Pesticides

**Figure 7:**
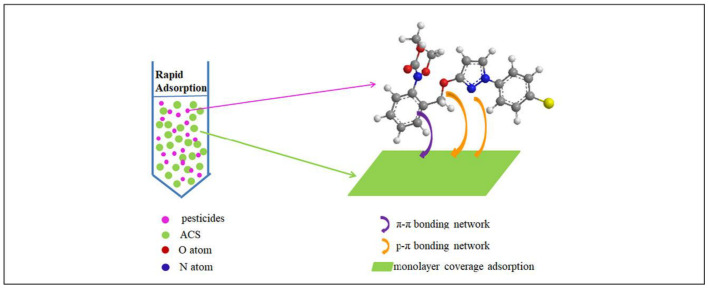
Application of mesoporous ACS to remove pesticides [86].

Given the possible risk to human health, pesticide poisoning of water has drawn a lot of attention. Many academics have recently shown interest in the removal of pesticides from water. Table 4 presents a list of starch-based adsorbents for pesticide removal. For the first time, mesoporous activated carbon from starch (ACS) was utilized to remove pesticides from water. Figure 7 illustrates the application of mesoporous ACS to remove pesticides. Scanning electron microscopy (SEM), FT-IR, X-ray photoelectron spectroscopy, and Brunauer–Emmet–Teller theory (BET) were used to investigate the mesoporous ACS, and it was shown to be highly efficient at cleaning water of contaminants. In fact, it removed 11 pesticides from water better than commonly used adsorbents, such as graphitized carbon black (GCB), activated carbon (AC), C18, and primary secondary amine (PSA) adsorbent. The inclusion of functional groups such as oxygen, nitrogen, and benzene ring bonds dramatically affected adsorption. This study contributes new information to the viability of using starch-based activated carbon as an effective pesticide adsorbent in water treatment [86]. 

Pesticides can be removed via a variety of techniques, including as adsorption, oxidation, enzymatic biodegradation, and photocatalytic degradation. Starch-derived mesoporous activated carbon adsorption is recognized as a highly effective method due to its low starting cost, ease of operation, flexibility, simplicity of design, and insensitivity to harmful pollutants. It is also one of the few methods capable of removing pollutants while remaining unaffected by them. Additionally, it is one of the few methods that can clear contaminants without being harmed by them, making it an extremely helpful tool [151]. Aside from this, starch has been investigated for its potential to be modified in order to achieve a balance between magnetism and van der Waals interaction on magnetic surfaces [152]. In one study, a magnetic starch material, known as Fe-starch@DABA-APTES, was synthesized by modifying starch with 3,5-diaminobenzoic acid and 3-aminopropyltriethoxysilane. This magnetic starch material was then utilized as an adsorbent for the extraction of organochlorine pesticides (OCPs) [150].

### 4.3. Removal of Heavy Metal Ions

Industrial wastewater and groundwater often contain various inorganic components, including arsenic (As), cadmium (Cd), chromium (Cr), cobalt (Co), copper (Cu), lead (Pb), mercury (Hg), nickel (Ni), zinc (Zn), and others [153]. Modified starches demonstrated substantial adsorption capabilities towards heavy metals by replacing hydroxyl groups with chemically active groups [55]. A wide range of starch derivatives with amino, amide, carboxyl and other groups were synthesized and used in water treatment [154]. Corn starch, for example, can be cross-linked and carboxymethylated to actively capture harmful divalent cations, which include Cu, Pb, Cd, and Hg ions present in water. By distributing 1% of the starch for a couple of minutes and then filtering the starch–metal complex, it was possible to efficiently remove about several hundred ppm of these metal ions from water at a low degree of substitution of carboxymethyl groups [57]. The starch can be easily restored through weak acidic washing, and the success of metal removal depends on avoiding highly acidic metal solutions. Increasing the levels of carboxymethylation and cross-linking can enhance the metal scavenging activity of starch, making it suitable for industrial applications [62,64,155,156,157].

Furthermore, starch can be used for the removal of heavy metal ions by grafting it with various vinyl monomers, including acrylic acid (AA), acrylic amide (AM), acrylonitrile, alkylmethacrylates, methylacrylonitrile, vinyl ketones, and 2-(dimethylamino) ethylacrylate. These polymers, despite their loosely crosslinked network structure and hydrophilic side groups, exhibit remarkable water absorption and retention capabilities [158]. In a study by Bai et al. [89], a cassava-starch-based copolymer was created by grafting AA and AM for the purpose of Cd(II) removal. This adsorbent demonstrated an adsorption capacity of 347.46 mg/g for Cd(II). Additionally, incorporating two or more polymers has become an increasingly significant strategy for the synthesis of novel biomaterials with improved properties that individual polymers could not achieve [154].

**Figure 8:**
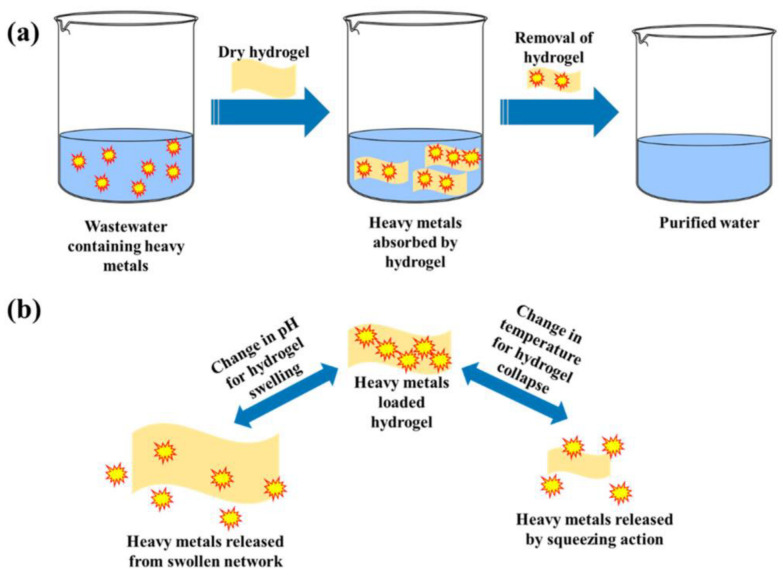
(**a**) The adsorption of heavy metals from wastewater using a starch-based hydrogel material and (**b**) Changes in structure as a result of external stimuli such as variations in pH and temperature [160].

Heavy metal ions were effectively eliminated by hydrogels with charged surfaces, one example being Chen et al. [159]’s Laponite RD (LRD) cross-linked hydrogels created by repeatedly freezing and thawing. Starch and polyvinyl alcohols were employed to create synthetic hydrogels (starch/PVA/LRD hydrogels), which were used to get rid of Cd(II). The LRD concentration in the hydrogels had a significant impact on the starch-based hydrogel’s capacity for adsorption. Strongly negative charges on the surface of LRD increased with an increase in LRD content in the hydrogel, which resulted in greater Cd(II) adsorption [159]. Figure 8a depicts a diagram for heavy metal removal from wastewater using a hydrogel-based adsorbent [160]. Upon changes in pH, temperature, etc., hydrogel adsorbents can release heavy metal ions, as illustrated in Figure 8b [160].

An alternative example is the utilization of a crosslinked carboxymethyl sago starch/citric acid (CMSS/CA) hydrogel for the adsorption of Pb(II), Cu(II), Ni(II), and Zn(II). The CMSS/CA hydrogel exhibited optimal adsorption capacities of 64.48 mg/g for Pb(II), 36.56 mg/g for Cu(II), 16.21 mg/g for Ni(II), and 18.45 mg/g for Zn(II) [62]. Chitosan, which contains several hydroxyl and amine functional groups, can be combined with various functional groups (carboxyl, hydroxyl, and amino groups) to produce carboxylmethyl chitosan (CMC). Hydrogel microspheres (HMs) were created by combining hydroxyethyl starch and carboxymethyl chitosan for the removal of Cd(II), Cu(II), and Ni(II). The maximum adsorption capacities were found to be 32.51 mg/g for Cd(II), 47.87 mg/g for Cu(II), and 27.18 mg/g for Ni(II) [53]. Additional examples of starch-based adsorbents for heavy metal ion removal are presented in Table 5.

### 4.4. Removal of Dye

The manufacturing processes of industries, like the leather, paper, and textile industries, release extremely dangerous and carcinogenic chemicals into wastewater. The release of waste dyes from textile finishing poses a significant threat to both natural water resources and human well-being. [83]. Polymers, and more specifically biopolymers, have a wide structure that affords several binding sites for dye molecules. This helps to neutralize the charge that the dye molecules carry, which in turn enables effective precipitation. In applications involving the coagulation of blood, biopolymers are the material of choice since, in contrast to traditional coagulants, they do not pose a threat to human health and have a lower impact on the environment [176].

Starch is a key component in enhancing the quality of the overall nanocomposite due to its amylose chains, which have a strong affinity for anionic dye molecules. A highly recommended alternative method for removing anionic-charged dyes involves using a mixture of starch, chitosan, and glutaraldehyde in specific proportions. This mixture works by utilizing the attractive properties of starch and chitosan to effectively remove the dyes [176]. The combination of starch and chitosan creates electrostatic and hydrophobic interactions that provide benefits in dye removal effects compared to just chitosan alone. These chitosan starch nanocomposites have the potential of 90% in the removal of anionic-charged dye through coagulation-flocculation [177,178,179].

As for cationic dyes such as methylene blue (MB), MNPs@Starch-g-poly(vinyl sulfate) nanocomposite can effectively remove such dyes. This nanocomposite showed excellent adsorption of MB with a capacity of 621 mg/g and could remove as much as 90% [180]. Additionally, radical polymerization can be used to create cassava-starch-based (CS-g-PAM) hydrogels using varied cassava starch contents and polyacrylamide (PAM). At increasing cassava starch concentrations, the hydrogels’ porous structure/pore size decreased. The CS-g-PAM hydrogel with 50% cassava starch showed an outstanding MB elimination adsorption capacity of 1917 mg/g [83]. Additionally, the incorporation of catecholamine functional groups onto starch-g-(acrylic acid-co-acrylamide) superabsorbent, a type of hydrogel, was achieved through the oxidative polymerization of dopamine (DA) for the purpose of adsorbing MB. A maximum adsorption capacity of 2276 mg/g was achieved at pH 9 within a 100 min timeframe [74].

**Figure 9:**
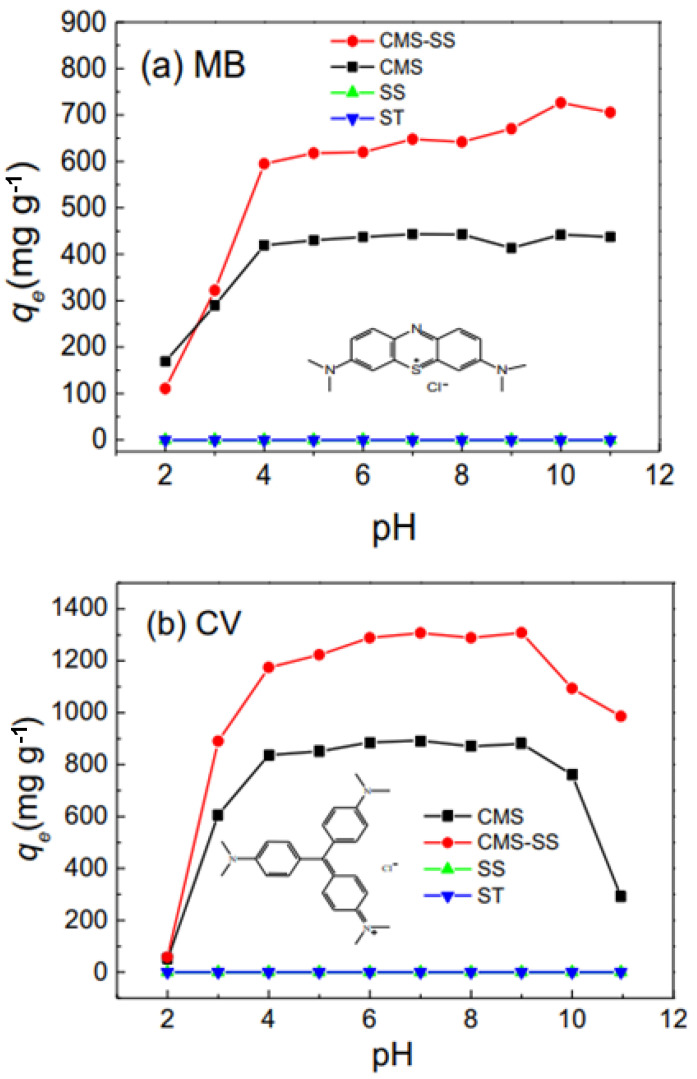
Effects of increased pH towards the adsorption process of CMS-SS, CMS, SS, and ST for cationic dye removal [161].

Another way of removing cationic dyes such as MB and crystal violet (CV) is by utilizing silica–sand/anionized-starch composite (CMS-SS). With effects such as pH, CMS-SS can produce a high yield of adsorption capacity towards MB and CV, as illustrated in Figure 9. This is because of the high efficiency of electrostatic interactions amongst the cationic pollutants with carboxylmethyl groups. As evident in the graph below, the increase in pH results in the staggering and stable increase of adsorption capacity of CMS-SS towards cationic dye pollutants. Even compared to other adsorbents, CMS-SS still shows superior quality in cationic dye removal [161].

CMS-SS is very recyclable and easy to recover. Due to its combination with silica–sand (SS), it is very easy to separate it from aqueous solutions. After being recovered from the first adsorption cycle of removing MB and CV, CMS-SS can still be utilized in adsorbing acid green 25 (AG25) anionic dyes [161]. This is because the surface charges were changed during the separation process. All in all, CMS-SS is much cheaper and has better resistance towards acid compared to other adsorbent materials, such as magnetic adsorbents [180,181]. Its rapid settling properties put it in a beneficial position and it is perfect for water treatment industries [182,183].

**Figure 10:**
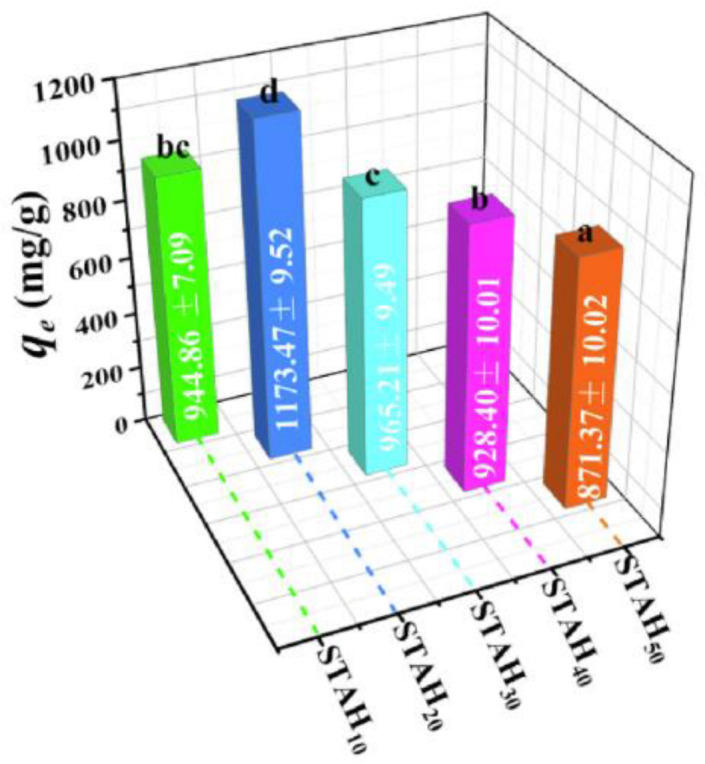
STAH_n_ equilibrium adsorption capability for MB [184]. Values followed by the same letters are not significantly different at *p* ≤ 0.05.

Starch-based high-performance adsorptive hydrogel (STAH) is another type of adsorbent suited for the removal of MB. Synthesized by grafting polyacrylic acid (PAA) onto starch, the process continues to crosslink with N,N′-methylene-bisacrylamide (MBA). STAH comes with different isomers. These isomers are STAH_10_, STAH_20_, STAH_30_, STAH_40_, and STAH_50_. Based on Figure 10, five of the STAH isomers showed great adsorption capability. Among five of them, STAH_20_ shows the best adsorption capacity of 2967.66 mg/g. STAH adsorbent can be reused more than three times [184]. More starch-based adsorbents for dye removal are presented in Table 6.

### 4.5. Removal of Pharmaceutical Pollutants

**Figure 11:**
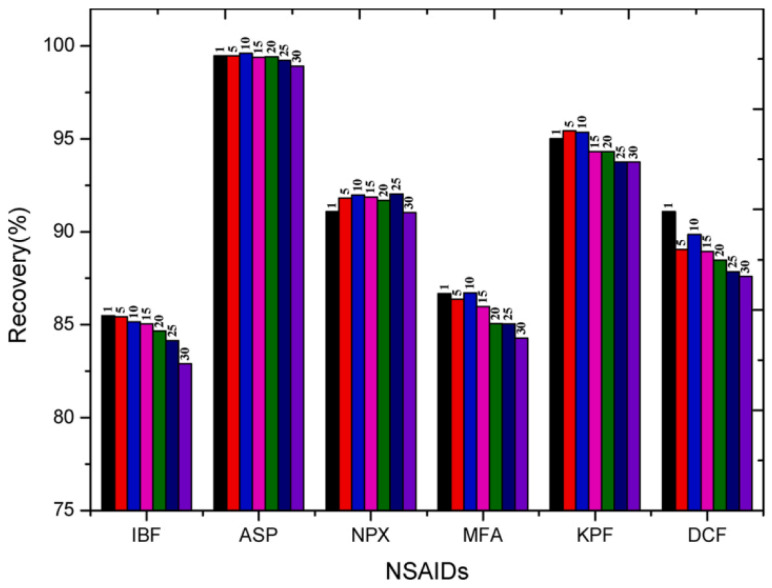
Recovery measurement of NSAIDs after every 5 reuse cycles of S-Mg/Al LDHs [196].

The discharge of trace amounts of pharmaceuticals into ecosystems is acknowledged as a serious environmental issue, resulting in persistent and immediate impacts on the environment [195]. Starch-Mg/Al-layered double hydroxide (S-Mg/Al LDH) is a synthesized composite utilized in the adsorption of non-steroidal anti-inflammatory drugs (NSAIDs) found in various water and wastewater sources. This adsorbent performs well due to its efficiency and high adsorption rate. S-Mg/Al LDH also showed good reusability performance when tested with optimized experimental parameters. As shown in Figure 11, the recovery for this adsorbent is at a high-end percentage. This is clearly a huge benefit for industries that aim for economical workflow [196]. Examples of starch-based adsorbents for pharmaceutical pollutants removal are listed in Table 7.

To remove tetracycline, carboxymethyl-starch-grafted magnetic bentonite [198], starch-stabilized magnetic nanocomposite [199], magnetic starch polyurethane polymer nanocomposite [200], and magnetic starch nanocomposite [201] were created. Shen et al. [198] compared corn-starch-grafted magnetic bentonite (SMB) to carboxymethyl-starch-grafted magnetic bentonite (CSMB) and discovered that the CSMB had a 28% higher tetracycline adsorption capacity compared to SMB. Regarding recyclability, the adsorption capacities of CSMB experienced a decrease of over 20% after the initial cycle due to the destructive effects of nitric acid treatment on some of the functional structures, resulting in a loss of adsorption capacity. After three cycles, the adsorption capacity of CSMB was reduced by 47% to 89.5 mg/g, compared to the initial capacity of 169.7 mg/g. On the other hand, SMB exhibited a slightly lower reduction in adsorption capacity after three cycles compared to CSMB. This indicates that SMB demonstrates better recyclability.

Other pharmaceutical pollutants, such as fluvastatin [197], dox [90], and bovine serum albumin [202], have also been investigated using starch-based adsorbents. The removal of fluvastatin can be accomplished using the magnetic MOF–starch hydrogel created by Mohamed and Mahmoud [197]. The magnetic MOF–starch hydrogel was developed via microwave irradiation and demonstrated several remarkable properties. It exhibits a maximum equilibrium adsorption capacity of 782.05 mg/g, a high surface area of 528.39 m^2^/g, a mesoporous structure with a pore size of 2.90 nm, and a highly crystalline structure. Within this system, three types of bonding are expected to occur. Firstly, there is H-bonding between carboxylic and OH groups, leading to physisorption. Secondly, covalent bond formation between carboxylic and OH groups facilitate ester formation, resulting in chemisorption. Lastly, coordinate bond formation occurs between the oxygen donor atoms in fluvastatin and the Zinc(II) ion in the magnetic MOF–starch hydrogel, leading to chemisorption [197].

On the contrary, carboxymethyl cassava starch (CMCS)-functionalized Fe_3_O_4_ magnetic nanoparticles were synthesized using a one-pot co-precipitation method for the removal of dox [90], whereas Fe_3_O_4_ magnetic nanoparticle-crosslinked gelatin–starch microspheres were prepared using a modified emulsion cross-linking method with glutaraldehyde as the cross-linking agent for bovine serum album removal [202]. The functionalized CMCS core structure swelled rapidly and had more carboxyl functional group sites for effectively adsorbing dox molecules [90]. Between the pH range of 4 to 9, the carboxyl groups underwent conversion into the negatively charged carboxylate form, while dox existed in a zwitterionic form. This conversion enhances the interaction between positively charged dox ions and the negatively charged CMCS-2@Fe_3_O_4_, thus increasing the efficiency of adsorption [203]. The interaction between carboxyl groups and dox molecules involves various intermolecular forces, such as H-bonding, π-interactions, and electrostatic interactions [90]. The adsorption process of dox by CMCS-2@Fe_3_O_4_ follows a multistep mechanism [204], primarily involving chemisorption through valency forces via electron sharing or exchange between CMCS-2@Fe_3_O_4_ and dox molecules [205], potentially accompanied by physical adsorption [90]. Initially, dox molecules rapidly transfer to the surface of CMCS-2@Fe_3_O_4_ through boundary layer diffusion. Subsequently, they continue to diffuse into the adsorptive sites within the pores of CMCS-2@Fe_3_O_4_ until the adsorption capacity reaches equilibrium [90].

Adsorbents made from rape straw biomass fiber/β-CD/Fe_3_O_4_ and β-cyclodextrin nanosponge (β−CD−M) have been proven to be effective in removing ibuprofen from water and sewage. The maximum adsorption capabilities of these adsorbents are 48.29 and 86.21 mg/g, respectively [128,129]. Ibuprofen can be removed from wastewater using β-CD-M with 84% to 100% efficiency for concentrations ranging from 2.06 to 0.021 mg L^−1^, respectively [129]. β−CD−M can be compared to a honeycomb, with the cells being β−CD molecules and the backbone consisting of elastic urethane and allophanate chains. When the β−CD−M is added to the ibuprofen solution, it is preorganized to allow the host to better suit the requirements of the guest molecules. It has a flat ring that is substituted with linear groups, which allows the molecule to be inserted into the β−CD cavity while also incorporating the functional groups in system stabilization. The complex is formed by all aromatic and even aliphatic protons [129]. The advantages of nanosponges include high adsorption efficiency despite a small surface area, non-toxicity, the potential for multiple regeneration and reuse, modeling of specific interactions, and low production and usage cost. However, large-scale application of β-cyclodextrin in wastewater treatment may be economically unfeasible due to its relatively expensive production cost.

Table 7 contains other examples of modified β−CD composite adsorbents. For instance, imipramine, bisphenol-S, procaine, ciprofloxacin [126], and acetaminophen [131] can all be removed with β−CD grafted chitosan. With an adsorption capacity of 200.86 mg/g, 0.01 g of calcium(II)-doped chitosan/-cyclodextrin composite adsorbent was able to remove 99.88% of acetaminophen from 20.0 mL of 20.0 mg/L aqueous solution at pH 7.2. Salbutamol and atenolol can be effectively removed using an electronegative silanized β-cyclodextrin adsorbent [132], while estradiol (E2) [133] and 17β-estradiol [134] can be removed using polyethersulfone nanofibers impregnated with β-cyclodextrin and β-cyclodextrin/poly (l-glutamic acid)-supported magnetic graphene oxide, respectively. An electronegative surface modified by introducing carboxyl groups via N-[(3-Trimethoxysilyl)propyl]ethylenediamine triaceticacid trisodium salt (EDTS) enables an electronegative silanized β-cyclodextrin adsorbent to effectively interact with various types of organic pollutants while maintaining excellent adsorption performance over a wide pH range. After modification with EDTS, there was a substantial increase in the maximum adsorption capacities of salbutamol and atenolol, which rose by 162% and 706% respectively, reaching values of 140.24 mg/g and 236.92 mg/g. These results highlight the potential for further advancements in the field of intelligent starch derivative-based adsorbents.

## 5. Outlook and Challenges

The most important challenges to using starch-based polymer materials as advanced adsorbents for sustainable water treatment is the production of sludge volume and its impact on the efficiency of water treatment. Starch-based materials, while suitable for sustainable water treatment, tend to produce lower sludge volume, which will slow the process of water treatment compared to chemical-based water treatment. Additionally, the high-cost implementation of starch-based materials is another challenge, as they require several modification processes before they can be used as adsorbents. Moreover, the application of starch-based materials on a large scale for water treatment poses a challenge, as they are currently only feasible for small-scale implementation. These challenges affect the overall effectiveness of starch-based materials for water treatment and limit the scale at which they can be used sustainably.

Starch is an organic and biocompatible material suitable for both ex situ and in situ water treatment as it is non-toxic and not harmful to living organisms. Second, starch-based adsorbents are abundant, biodegradable, and renewable. Starch-based adsorbents can be regenerated and reused until the end of their lifetime. These adsorbents are safely decomposed by bacteria or other living organisms or disposed of through incineration and landfilling. Prior to disposal, non-biodegradable pollutants can be removed using desorption. Desorption requires the use of chemical reagents, which raises the cost; however, the use of starch-based adsorbents reduces pollution and solves waste disposal issues. Therefore, starch-based adsorbents hold significant potential to be used as eco-friendly materials for sustainable water treatment.

## 6. Concluding Remarks and Future Perspectives

The effective use of starch for wastewater treatment comes from its vast availability and sustainability as a natural polysaccharide. In wastewater treatment applications, modified starch products have showed promise in the removal of a range of contaminants, including oil, organic solvents, pesticides, heavy metal ions, dyes, and pharmaceutical pollutants. An example of innovative use of starch-based materials is the development of raspberry-like starch-based polymer microspheres. These microspheres are created through Pickering polymerization and grafting of poly(ethylene imine) (PEI) onto amino-functionalized composite particles. These microspheres not only have the capability to separate oil and water, but they also exhibit simultaneous removal of Cr(VI) and Indigo carmine. The efficiency of oil and water separation is influenced by the dosage of PEI. The resulting composite particles possess unique characteristics, such as rough structures, distinctive surface wettability, and positive charge. This combination enables them to simultaneously separate water-in-oil (W/O) and oil-in-water (O/W) emulsions within a specific dosage range of PEI. Moreover, the amino-functionalized composite particles carry a positive charge, which enhances their ability to effectively absorb anionic water-soluble pollutants. The removal rate of these pollutants during the oil/water separation process can reach nearly 90%.

While there are promising applications for starch-based adsorbents in environmental wastewater treatment, there is still considerable potential for future advancements in the development of intelligent adsorbents based on starch derivatives. The utilization of a trifunctional chitosan-EDTA-β-cyclodextrin adsorbent allows for the simultaneous removal of metals and organic micropollutants. This adsorbent demonstrated a monolayer adsorption capacity of 0.803 mmol g^−1^ for Pb(II) and 1.258 mmol g^−1^ for Cd(II), while exhibiting a heterogeneous adsorption capacity of 0.177 mmol g^−1^ for bisphenol-S, 0.142 mmol g^−1^ for ciprofloxacin, 0.203 mmol g^−1^ for procaine, and 0.149 mmol g^−1^ for imipramine, respectively. Further research is necessary to enhance the adsorption properties of starch- and starch-derivative-based adsorbents, optimize their adsorption capacity, and investigate methods for regeneration and reuse while minimising the reduction in adsorption capacity. Additionally, the potential applications of starch-based adsorbents can be expanded beyond wastewater treatment. Industries such as food and packaging, pharmaceuticals, and others could benefit from the utilization of starch-based adsorbents. Further exploration and discovery in this area are warranted to uncover new possibilities and opportunities. 

## Figures and Tables

**Table 1:** Comparison of adsorption capacity between starch-based and conventional adsorbents.

Type of Adsorbent	Type of Pollutant	Pollutant Concentration (mg/L)	Adsorption Capacity (mg/g)	Ref.
Starch-functionalized Iron(III) oxide (Fe_2_O_3_) nanoparticles	Lead (II)	150–450	2000	[64]
Activated carbon functionalized magnetic iron oxide nanoparticles		10–100	61.82	[65]
Activated carbon		50	30	[66]
Amine functionalized Fe_3_O_4_ magnetic nanoparticle dialdehyde starch	Mercury (II)	150	318.87	[59]
Activated carbon		-	138	[67]
Starch-based amino-functionalized microspheres	Chromium (VI)	50	734.8	[60]
Activated carbon		200	145	[68]
Starch-g-polyacrylamide/Fe_3_O_4_/graphene oxide nanocomposite	Nickel (II)	20	290	[69]
Activated carbon prepared from coir pith		20	62.5	[70]
Starch derived zinc carbon foam-like	Malachite green (MG)	25–100	1200	[71]
Carbon prepared from waste jack fruit peel		20–60	166.37	[72]
Starch-based amino-functionalized microspheres	Indigo carmine (IC)	50	423.69	[60]
Activated carbon		-	16.3–77.7	[73]
Starch-g-(acrylic acid-co-acrylamide) functionalized catecholamine	Methylene blue (MB)	2700	2276	[74]
Refused derived fuel		100	83	[75]

**Table 2:** Starch-based adsorbents for in situ water remediation.

Starch-Based Adsorbents	Pollutants	In Situ Remediation		Ref.
		Adsorption (%)	Desorption (%)	
Corn starch without pretreatment	Betalain	36.40	35.20	[93]
Corn starch with methanol pretreatment		21.80	58.30	
Wheat starch without pretreatment		20.50	75.01	
Wheat starch with methanol pretreatment		32.72	44.40	
Soluble starch and facultative psychrophilic denitrifier immobilized on macro-porous cellulose	Nitrate	99.5	-	[94]

**Table 3:** Starch-based adsorbents for oil and organic solvent removal.

Starch-Based Adsorbents	Pollutants	Adsorption Capacity (mg/g)	Ref.
Sweet potato/Corn/Starch-based adsorbents	Ethanol	150	[85]
Superhydrophobic starch-based adsorbent	Chloroform	7560	[140]
	n-hexane	2500	
Superhydrophobic starch/iron oxide (Fe_3_O_4_)/silylated silicon dioxide (SiO_2_) nanoparticles/cryogel	Chloroform	7780	[141]
	n-hexane	2720	
Rice straw-cationic starch aerogel	Oil	13,000	[142]
Superhydrophobic/oleophilic starch cryogel	Chloroform	7530	[143]
	n-hexane	2610	
Superoleophilic starch-based cryogels coated by silylated porous starch/Fe_3_O_4_ hybrid micro/nanoparticles	Chloroform	7570	[144]
	n-hexane	2590	
Magnetic modular cryogel	Chloroform	6190	[145]
	n-hexane	2060	
Starch derived zinc carbon foam-like	Castrol 2T	2937%	[71]
	Gear oil	2375%	
Starch-graft-styrene hypercrosslinked polymers	Acetophenone,	93.6%	[146]
	1-phenylethanol	74.4%	
Starch-based amino-functionalized microspheres	Oil/water separation	99.9%	[60]
Sweet potato/Corn/Starch-based adsorbents	tert-butyl alcohol (TBA), isopropanol, ethanol	-	[91]
Sweet potato/Corn/Starch-based adsorbents	TBA	-	[92]

**Table 4:** Starch-based adsorbents for pesticide removal.

Starch-Based Adsorbents	Pollutants	Adsorption Capacity (mg/g)	Ref.
Corn/starch-based mesoporous activated carbon (ACS)	pyraclostrobin	66.2	[86]
Microporous maize starch immobilized laccase	atrazine	0.2527	[148]
	prometryn	0.1323	
P-doped biochar from corn straw	triazine	79.6	[149]
Iron-starch modified with 3,5-diaminobenzidine and (3-aminopropyl) triethoxysilane (Fe-starch@DABA-APTES)	Endosulfan	0.00025–0.00200	[150]
	Heptachlor	0.00001–0.00075	
	Aldrin	0.00001–0.00075	
	Isobenzan	0.00001–0.00075	
	Chlordane	0.00010–0.00100	
	Dieldrin	0.00001–0.00075	
	Endrin	0.00010–0.00100	

**Table 5:** Starch-based adsorbents for heavy metal ion removal.

Starch-Based Adsorbents	Pollutants	Adsorption Capacity (mg/g)	Ref.
Starch–chitosan-based hydrogel microspheres	Cd(II)	32.51	[53]
	Cu(II)	47.87	
	Ni(II)	27.18	
Starch-functionalized iron(III) oxide (Fe_2_O_3_) nanoparticles	Pb(II)	2000	[64]
	Hg(II)	133.3	
	Cd(II)	322.58	
Crosslinked carboxymethyl sago starch/citric acid hydrogel	Pb(II)	64.48	[62]
	Cu(II)	36.56	
	Ni(II)	16.21	
	Zn(II)	18.45	
Walnut shell ash/starch/iron oxide (Fe_3_O_4_)	Cu(II)	45.4	[95]
Silica-sand/anionized-starch composite	Cu(II)	383.08 ± 13.50	[161]
Starch/Fe_3_O_4_-g-p(AA-r-HEMA)	Cu(II)	75.5	[162]
Magnetic starch-g-polyamidoxime/montmorillonite/Fe_3_O_4_ nanocomposites	Cu(II)	163	[163]
Starch-based amino-functionalized microspheres	Cr(VI)	734.8	[60]
Starch-crosslinked magnetic ethylenediamine	Cr(VI)	210.7	[164]
Starch-functionalized iron oxide nanoparticles	Cr(VI)	9.02	[165]
Polyethyleneimine-modified magnetic starch microspheres (PEI/MSMs)	Cd(II)	187.00	[166]
Magnetic starch microspheres (AAM- MSM)	Cd(II)	39.98	[167]
Cassava-starch-grafted copolymerized AA and AM	Cd(II)	347.46	[89]
Eggshell/starch/Fe_3_O_4_ nanocomposite	Cd(II)	48.54	[168]
	Pb(II)	57.14	
Starch-stabilized magnetic nanoparticles	Ni(II)	-	[169]
Starch-g-polyacrylamide/Fe_3_O_4_/graphene oxide nanocomposite	Ni(II)	290	[69]
Dialdehyde cornstarch	Gold(III)	298.5	[170]
Polyethylene-g-poly (acrylic acid)-co-starch/organo-montmorillonite hydrogel	Pb(II)	430	[61]
Starch graft poly(acrylic) acid	Pb(II)	118.61	[171]
Starch graft poly(acrylonitrile)		115.83	
Amine-functionalized Fe_3_O_4_ magnetic nanoparticle dialdehyde starch	Hg(II)	318.87	[59]
Magnetic starch/polyethyleneimine	Hg(II)	244.87	[153]
Starch-functionalized maghemite nanoparticles	As(III)	8.88	[172]
CO_2_-assisted modified magnetic starch-Fe_3_O_4_ nanoparticles	As(III)	124	[173]
Starch-functionalized magnetite nanoparticles	As(III)	68.3	[174]
	As(V)	74.8	
Starch-bridged magnetite nanoparticles	As(V)	248	[175]
Carboxymethyl starch-g-polyvinyl imidazole	Cu(II)	83.6	[154]
	Cd(II)	53.2	
	Pb(II)	65	

**Table 6:** Starch-based adsorbents for dye removal.

Starch-Based Adsorbents	Pollutants	Adsorption Capacity (mg/g)	Ref.
Hydrogel microspheres	Methylene blue (MB)	106.97	[53]
	Eosin yellow (EY)	143.55	
Zinc–starch and zerovalent iron extrudates	MB	61.03	[185]
Magnetism carboxymethyl starch/poly(vinyl alcohol) gel	MB	23.53	[186]
Zinc–starch–metal–organic coordination polymers-Fe_3_O_4_ NPs composite	MB	37.42	[187]
Double-cross-linked amphoteric hydrogel	MB	133.65	[188]
	Congo red (CR)	64.73	
Starch derived zinc carbon foam-like	Crystal violet (CV)	25,000	[71]
	Malachite green (MG)	1200	
	CR	1428.57	
Cationic tapioca starch (CTS)-functionalized magnetic nanoparticles (CTS@Fe_3_O_4_)	Caffeic acid (CA)	185	[189]
	Gallic acid (GA)	160	
	Melanoidin (ME)	580	
Rice starch	Methyl orange (MO)	173.24	[88]
Graham starch	MO	151.27	
Cassava starch-based hydrogels grafted polyacrylamide	MB	2000	[83]
Starch-g-(acrylic acid-co-acrylamide)-functionalized catecholamine	MB	2276	[74]
Starch–magnesium/aluminum-layered double hydroxide	Amaranth	665	[190]
	Tartrazine	186	
	Sunset yellow (SY)	71	
	EY	65	
Clinoptilolite/Starch/CoFe_2_O_4_	MB	31.81	[191]
	Methylene violet (MV)	31.15	
	CV	32.84	
Corn starch magnetic carbonaceous adsorbent	MV	344.92	[78]
Carboxymethyl starch-g-polyvinyl imidazole	CR	83.66	[154]
	CV	91.58	
Silica-sand/anionized-starch composite	MB	653.31 ± 27.30	[161]
	CV	1246.40 ± 34.10	
Clay/starch/MnFe_2_O_4_	SY	67.82	[192]
	Nile blue (NB)	72.25	
Magnetic nanoparticles@starch-g-poly(vinyl sulfate) nanocomposite	MB	621	[180]
	MG	567	
Starch-coated Fe_3_O_4_ magnetic nanoparticles	Option Blue (OB)	128.83	[193]
Magnetic starch-based composite hydrogel microspheres	MB	64.05	[181]
Starch-functionalized multiwall carbon nanotube composites	MO	135.8	[194]
Starch-based amino-functionalized microspheres	Indigo carmine (IC)	423.69	[60]

**Table 7:** Starch-based adsorbents for pharmaceutical pollutants removal.

Starch-Based Adsorbents	Pollutants	Adsorption Capacity (mg/g)	Ref.
Magnetic metal–organic frameworks (MOFs)-starch hydrogel	Fluvastatin	782.05	[197]
Carboxymethyl-starch-grafted magnetic bentonite	Tetracycline	169.7	[198]
Starch-stabilized magnetic nanocomposite	Tetracycline	24.194	[199]
Magnetic starch polyurethane polymer	Tetracycline	19.272	[200]
Magnetic starch nanocomposite	Tetracycline	8.79	[201]
Carboxymethyl cassava starch (CMCS)-functionalized Fe_3_O_4_ magnetic nanoparticles	Doxorubicin hydrochloride (Dox)	235.17 ± 1.75	[90]
Fe_3_O_4_ magnetic nanoparticles crosslinked gelatin-starch microspheres	Bovine serum album	120	[202]
Rape straw/β-CD/Fe_3_O_4_	Ibuprofen	48.29	[128]
β-Cyclodextrin nanosponge (β−CD−M)	Ibuprofen	86.21	[129]
Nanocomposite adsorbent based on β-cyclodextrin-PVP-clay	Naproxen	3.46	[130]
Bio-derived chitosan-EDTA-β-cyclodextrin (CS-ED-CD) trifunctional adsorbent	Bisphenol-S Ciprofoxacin Procaine Imipramine	43.66 47.11 47.98 41.94	[126]
Calcium(II)-doped chitosan/β-cyclodextrin composite	Acetaminophen	200.86	[131]
Electronegative silanized β-cyclodextrin adsorbent	Salbutamol Atenolol	140.24 236.92	[132]
Polyethersulfone nanofibers impregnated with β-cyclodextrin	Estradiol (E2)	0.000115–0.00029	[133]
β-cyclodextrin/poly (l-glutamic acid) supported magnetic graphene oxide	17β-estradiol	85.5	[134]

## Data Availability

No new data were created or analyzed in this study. Data sharing is not applicable to this article.
